# In Vivo Enzyme Catalytic Rates in Formate‐Growing *Methanococcus maripaludis*

**DOI:** 10.1111/1751-7915.70417

**Published:** 2026-07-24

**Authors:** Celma Mekki, Hamza Faquir, Enrique de Dios Mateos, Silvan Scheller, Paula Jouhten

**Affiliations:** ^1^ Department of Bioproducts and Biosystems, School of Chemical Engineering Aalto University Espoo Finland

## Abstract

Turnover numbers ($k_{\mathrm{cat}}$) describe intrinsic catalytic capacities of enzymes. Although they have been characterized in vitro for many model organisms, such data is scarce for anaerobic Archaea like the methanogen *Methanococcus maripaludis* . Moreover, the apparent in vivo catalytic rates of enzymes operating in C1 utilization have neither been quantified experimentally nor predicted computationally. Here, we determined the in vivo catalytic rates of 99 *M. maripaludis* enzymes during growth on formate as the sole carbon source and electron donor. The determination was performed using previously published proteomics data and genome‐scale metabolic model simulations constrained with experimental data across multiple growth rates. The obtained in vivo catalytic rates ($k_{\mathrm{app}}$) were compared to sequence‐derived maximum turnover numbers ($k_{\mathrm{cat}}$) previously predicted by a machine‐learning model trained on in vitro data. Sequence‐derived turnover numbers and maximum in vivo catalytic rates did not correlate. The quantitative insight into methanogenic C1 metabolism and the in vivo catalytic rates can be used to guide metabolic engineering strategies. Engineered methanogens can serve as hosts in disruptive solutions of future biotechnological chemical production from one‐carbon compounds.

## Graphical abstract

In vivo catalytic rates of *Methanococcus maripaludis* enzymes during C1 carbon source limited growth were determined from metabolic model estimated fluxes and proteomics data. They showed weak agreement with turnover numbers predicted from sequences. The low enzyme saturation, rather than thermodynamic limitations, primarily reduced the catalytic performance.

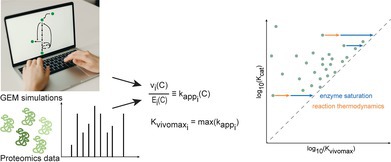

## 1 Introduction

Archaea are key players in global biogeochemical cycles and help shape greenhouse gas emissions (Offre et al. 2013; Wang et al. 2025). They are important contributors to the global carbon cycle (Könneke et al. 2014; Borrel et al. 2016). Anaerobic methanotrophic archaea (ANME) that are phylogenetically related to methanogens are present in marine sediments and carry out the reverse process, thus helping to prevent the escape of methane to the atmosphere (Valentine and Reeburgh 2000). In addition to this CO_2_ to methane metabolism, some methanogens are also able to use diverse substrates, including one‐carbon compounds such as CO, formate, methanol, methylamines, methyl sulfides, acetate, or even coal for their energy metabolism (Rother and Metcalf 2004; Mayumi et al. 2016). Despite these differences, all pathways converge on the reduction of methyl‐coenzyme M to methane, a reaction catalysed by methyl‐coenzyme M reductase (Mcr), the key enzyme responsible for biological methane formation (Rother and Metcalf 2004, Mayumi et al. 2016).

Besides their importance for global carbon cycling and being a contributor to global warming, methanogens enable several applications for biotechnology that can help mitigate global warming. Communities of methanogens catalyse the final steps in anaerobic digestions, where organic material is converted to produce biogas (i.e., a mixture of methane and CO_2_), which can be utilized for electricity production or as a transportation fuel (Weiland 2010). Overall, methanogens convert renewable or waste carbon into methane. Hydrogenotrophic methanogens enable the conversion of CO_2_ to methane with the help of renewable hydrogen. This process is being commercialized, e.g., to convert biogas to gas‐grid quality methane, whereby methanogenic communities or via pure strains of *Methanothermobacter* are utilized as biocatalysts. Future applications of methanogens may include metabolic engineering to co‐produce value‐added products, such as terpenes (Mühling et al. 2024; Mentrup et al. 2025) while producing methane, or even engineering methanogens to produce value‐added products instead of methane.

*M. maripaludis* is a model organism for hydrogenotrophic methanogenesis and archaeal genetics because it grows fast (i.e., with a doubling time of ~2 h) at 38°C, it grows on minimal media (Whitman et al. 1986), is capable of fixing nitrogen (Zellner and Winter 1987; Kessler et al. 2001; Haydock et al. 2004), has a small and sequenced genome (Hendrickson et al. 2004; Poehlein et al. 2018) and it is genetically tractable (Hendrickson et al. 2004). *M. maripaludis* naturally reduces CO_2_ to methane concomitant with energy conservation, via the same steps that are also utilized for the catabolism in *Methanothermobacter* species that are currently utilized industrially for biomethanation. CO_2_ is fixed in this organism via a modified reductive acetyl‐CoA (Wood–Ljungdahl) pathway (Can et al. 2023) that does not require direct ATP investment, which makes methanogens an interesting host organism for energy‐efficient CO_2_‐valorization. Although growth typically requires H_2_ as electron donor, this methanogen can also use formate as both carbon and electron sources via multiple formate dehydrogenases, providing metabolic flexibility and altering yields and regulation compared with H_2_‐based growth (Costa et al. 2013; Xue et al. 2025). Appealingly, the advancement of CRISPR‐Cas9 and Cas12a tools (Bao et al. 2022; Li et al. 2023) enable engineering of *M. maripaludis* for chemical production beyond methane.

Despite *M. maripaludis* being a genetically tractable model methanogen, its enzymatic and physiological parameters are poorly characterized. For instance, experimentally in vitro determined enzyme turnover numbers ($k_{\mathrm{cat}}$) remain limited to broadly investigated organisms like *Escherichia coli* . Determining the values of kinetic parameters $k_{\mathrm{cat}}$ and $K_{M}$ for *M. maripaludis* is also challenging because of the high oxygen‐sensitivity of the enzymes, the metallo‐enzymes in particular. Hence, it is often not clear whether in vitro experiments show full activity or whether the enzymes are only partially active in vitro. Furthermore, the high diversity of methanogens leads to spread out work on different species, which hinders comprehensive characterization of any. e.g., enzymatic studies have been performed in wildtype methanogens such as *Methanosarcina barkeri* and *Methanothermobacter marburgensis* , while enzymatic data is scarce for *M. maripaludis* .

Here, the lack of enzyme kinetic data on *M. maripaludis* was addressed by determining the apparent in vivo catalytic rates from proteomics and flux data with a computational approach previously demonstrated for *E. coli* (Davidi et al. 2016) and yeast *Saccharomyces cerevisiae* (Chen and Nielsen 2021). Fluxes of intracellular reactions were determined by performing simulations of *M. maripaludis* iMR539 genome‐scale metabolic model (GEM) (Richards et al. 2016) constrained with experimental extracellular flux data. The experimental data, including quantitative proteomics, were obtained from Müller et al. (2021) from *M. maripaludis* growing on formate in chemostat cultures at different dilution rates. We further compared the obtained in vivo catalytic rates of enzymes to turnover numbers retrieved from Metabolic Atlas (Lyu et al. 2025) that had been previously predicted from in vitro data using a deep learning model (Li et al. 2022).

## 2 Results

### 2.1 Estimation of Energy Expenditure to Maintenance

In order to accurately estimate the intracellular fluxes needed for the characterization of the apparent in vivo catalytic rates of *M. maripaludis* enzymes, the maintenance energy parameters of the *M. maripaludis* GEM iMR539 (Richards et al. 2016) were first determined. To correctly model the energy expenditure to maintenance during growth on formate, the growth‐associated maintenance energy (GAM) and the non‐growth‐associated maintenance energy (NGAM) parameters were determined from experimental data (Müller et al. 2021). GAM accounts for the energy (i.e., in the form of ATP) demand for maintenance functions tied to growth, whereas NGAM represents the energy usage for maintaining a living state of cells. We estimated GAM and NGAM using previously published physiological data on *M. maripaludis* growing in chemostat cultures on formate at four different dilution rates (with three biological replicate cultures at each dilution rate) (Müller et al. 2021). For estimates of each of the 12 cultures, formate utilization rate and growth rate were set to experimentally determined values, and ATP hydrolysis was maximized. The slope of maximized ATP hydrolysis flux as a function of growth rate yielded a growth‐associated maintenance (GAM) of 51.10 mmol/gCDW (Figure S1A). By grid search minimizing the total squared error between genome‐scale metabolic model predicted and measured growth rates gave NGAM of 0.83 mmol/(g CDW h) (Figure S1B). Using these GAM and NGAM parameter values, the model‐predicted methane excretion closely followed the experimentally determined methane formation in the chemostat cultures with an approximately linear dependence on growth rate (Figure S1C). Further, model simulations reproduced the experimentally observed relationship between growth rate and formate utilization rate across chemostat cultures (Figure S1D).

### 2.2 Computing the Apparent In Vivo Catalytic Rates

**FIGURE 1:**
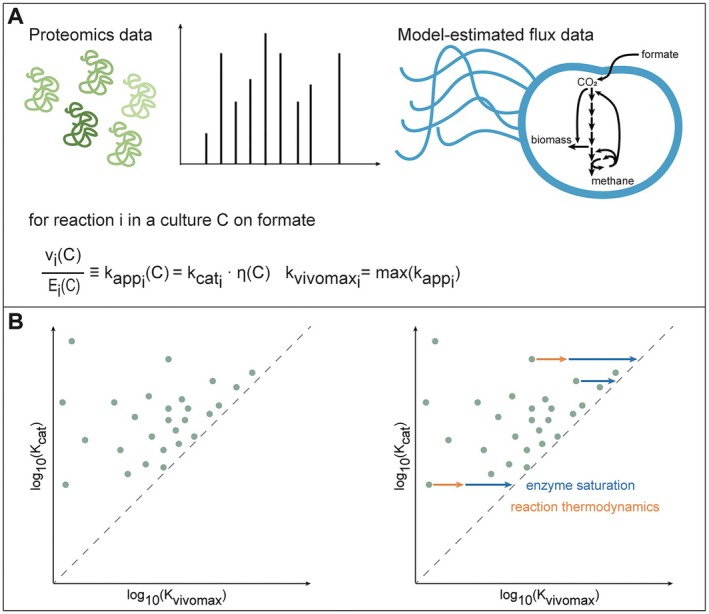
Workflow for characterizing in vivo catalytic rates of metabolic enzymes of *M. maripaludis*. (A) Schematic overview of determining the in vivo enzyme catalytic rates of *M. maripaludis* growing on formate from quantitative proteomics and fluxes estimated by performing genome‐scale metabolic model simulations constrained with physiological data across dilution rates in chemostat. *E*_*i*_ (*C*) refers to the concentration of an enzyme catalysing reaction i in culture *C* on formate, *v*_*i*_ (*C*) refers to the flux of reaction i in culture *C* on formate, $k_{{\mathrm{app}}_{i}}$ (*C*) refers to the apparent in vivo catalytic rate of an enzyme catalysing reaction i in culture *C* on formate, $k_{{\mathrm{cat}}_{i}}$ refers to the turnover number of an enzyme catalysing reaction I and *η* is a function ranging between 0 and 1 describing the deviation of the in vivo catalytic rate from the turnover number, due to condition‐dependent factors e.g., reaction thermodynamics and enzyme saturation. $k_{{\text{vivomax}}_{i}}$ refers to the maximum $k_{{\mathrm{app}}_{i}}\;\left(C\right)$ across cultures on formate. (B) Comparative analysis of in vivo catalytic rates and $k_{\mathrm{cat}}s$ predicted from sequence by machine learning from Metabolic Atlas (Lyu et al. 2025) (left). Limited enzyme saturation and thermodynamics likely deviate $k_{\text{vivomax}}$ from $k_{\mathrm{cat}}$ (right).

The GAM and NGAM parameterized *M. maripaludis* GEM iMR539 (Richards et al. 2016) was next simulated for each of the 12 cultures by using the extracellular experimental fluxes from the chemostat cultures of *M. maripaludis* on formate (Müller et al. 2021) as constraints to estimate the corresponding fluxes of intracellular reactions. With the estimated flux data and experimental proteomics data (Müller et al. 2021) of *M. maripaludis* growing on formate the determination of the apparent in vivo catalytic rates of enzymes, $k_{\mathrm{app}}s$, was performed following the approach by Davidi et al. (2016) (Figure 1). For a reaction catalysed by a single enzyme, $k_{\mathrm{app}}$ in a given culture C was defined as $v\left(C\right)/E\left(C\right)$. This quantity reflects the realized catalytic activity in the specific cultures and relates to the intrinsic turnover number through $k_{\mathrm{app}}\left(C\right)=k_{\mathrm{cat}}\cdot \eta \left(C\right)$, where *η*(*C*) is a function (0 < *η*(*C*) ≤ 1) representing the culture‐specific reduction in catalytic rate due to e.g., enzyme saturation or backward flux (Davidi et al. 2016). To ensure interpretability, the determination of the in vivo catalytic rates was focused on reactions catalysed by unique homomeric enzymes, excluding reactions involving multiple isozymes or heteromeric complexes where active‐site assignment is uncertain. This list of reactions was further filtered for fluxes that could have substantially varying values (i.e., flux range width between minimum and maximum values > 100). For each enzyme, the highest $k_{\mathrm{app}}$ value observed across all conditions (i.e., all chemostat cultures at different dilution rates) was taken as its maximal in vivo catalytic capacity, $k_{\text{vivomax}}$ (Figure 1). The $k_{\text{vivomax}}$ was successfully determined for 99 monomeric enzymes of *M. maripaludis* and they ranged from 0.005 to 73.3 s^−1^.

### 2.3 Correlation Between In Vivo Catalytic Rates and Sequence Derived $k_{\mathrm{cat}}$ Values

**FIGURE 2:**
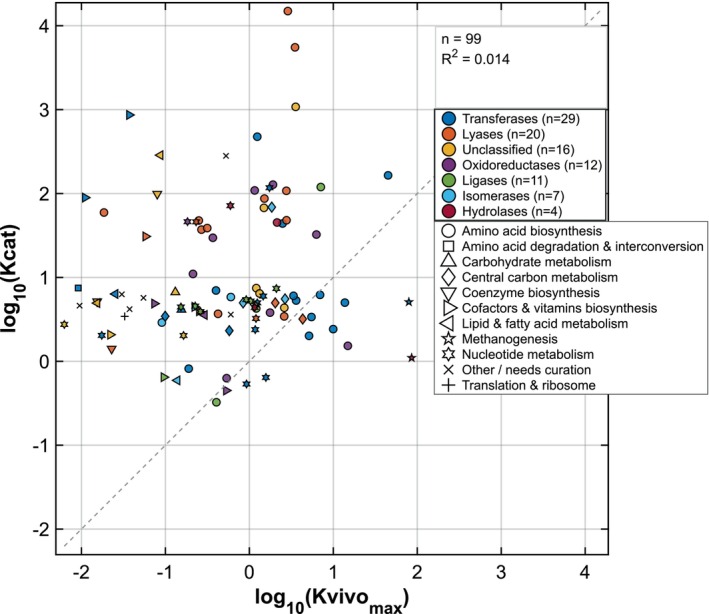
Computed $k_{\text{vivomax}}$ and $k_{\mathrm{cat}}$ values of metabolic enzymes in *M. maripaludis*. A log–log plot of computed $k_{\text{vivomax}}$ and $k_{\mathrm{cat}}$ values of 99 metabolic enzymes where the shapes visualize the processes in which the enzymes are involved, and colours indicate the types of enzymes. Number of enzymes n and Pearson correlation coefficient *R* ^2^ are shown in upper right corner.

Next, we compared the determined 99 maximal in vivo catalytic rates ($k_{\text{vivomax}}$) of *M. maripaludis* monomeric enzymes to sequence‐based turnover numbers ($k_{\mathrm{cat}}$) from Metabolic Atlas (Lyu et al. 2025) originating from deep learning model trained with in vitro data (Li et al. 2022) (Table S1). Computed $k_{\text{vivomax}}$ values and sequence derived $k_{\mathrm{cat}}$s were not correlated (*R* ^2^ = 0.0144) (Figure 2). The $k_{\text{vivomax}}$ values were predominantly reduced compared to $k_{\mathrm{cat}}$s which is consistent with $k_{\text{vivomax}}$ reflecting not only the intrinsic turnover number but also the condition‐dependent phenomena such as substrate saturation and reaction thermodynamics (Davidi et al. 2016).

**FIGURE 3:**
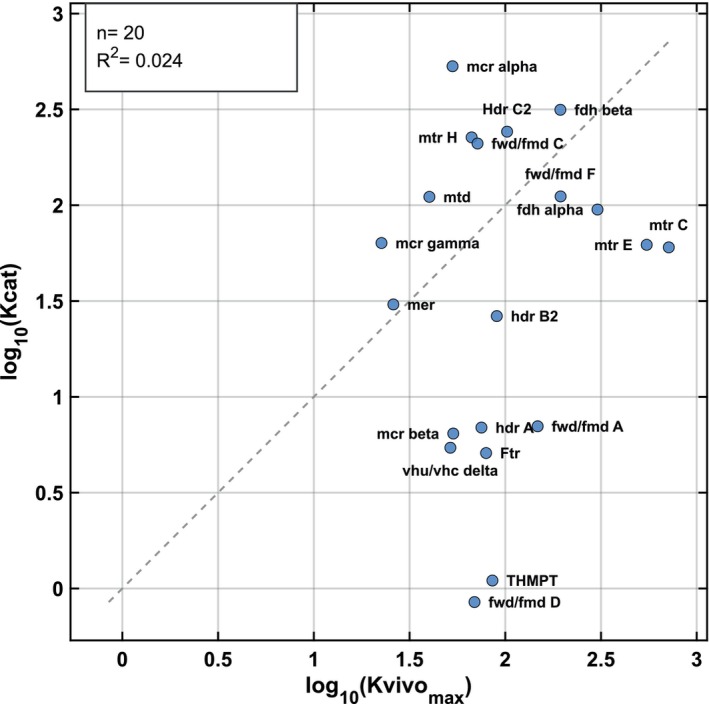
Computed $k_{\text{vivomax}}$ and $k_{\mathrm{cat}}$ across growth conditions for enzymes involved in energy metabolism. Log–log plot of $k_{\text{vivomax}}$ and $k_{\mathrm{cat}}$ of enzymes (including subunits of multimeric enzymes) involved in the energy metabolism of *M. maripaludis* (single‐polypeptide enzymes: Ftr, Mtd, Mer; complexes: McrA/McrB/McrG, MtrE/MtrC/MtrH, FdhA/FdhB, HdrA/HdrB2/HdrC2, Fwd/Fmd (A, C, D, F) and Vhu/Vhc (α, γ, δ)) when *M. maripaludis* grew on formate in chemostat cultures (Müller et al. 2021). Number of enzymes or enzyme subunits n and Pearson correlation coefficient *R* ^2^ are shown in upper right corner.

Several lyase $k_{\text{vivomax}}$ values were notably reduced from the corresponding $k_{\mathrm{cat}}$ values, whereas transferase values tended to be found closer to parity between $k_{\text{vivomax}}$ and $k_{\mathrm{cat}}$ (Figure 2). This suggested that catalytic mechanism could influence the condition‐dependency of catalytic rates. Interestingly, in contrast to most others, enzymes involved in methanogenesis were found with $k_{\text{vivomax}}$ near to $k_{\mathrm{cat}}$ values, potentially even exceeding them. $k_{\text{vivomax}}$ may surpass $k_{\mathrm{cat}}$ values when the catalytic capacities are underrated due to poor representation in the training datasets (Li et al. 2022), or when the in vitro experiments underestimate the catalytic capacities of enzymes. Underestimation may occur when enzyme activities are sensitive to experimental conditions like presence of oxygen. Several enzymes involved in methanogenesis, such as methyl‐coenzyme M reductase (Thauer 2019), lose their activity rapidly in presence of even trace amount of oxygen (Singh et al. 2003; Costa and Whitman 2023) and are underrepresented in databases. Further, to characterize in vivo catalytic rates versus $k_{\mathrm{cat}}s$ for energy metabolism, $k_{\text{vivomax}}s$ were separately estimated for subunits of multimeric enzymes involved in energy metabolism of *M. maripaludis* (i.e., methyl‐H4MPT:HS‐coenzyme M methyltransferase, methyl‐coenzyme M reductase, formate dehydrogenase, formylmethanofuran dehydrogenase, heterodisulfide reductase and F420‐non‐reducing [NiFe] hydrogenase) which suffers in from not accounting for complex stoichiometry and assembly into an active enzyme. Together with single‐polypeptide enzymes (i.e., formylmethanofuran:H4MPT formyltransferase, F420‐dependent methylenetetrahydromethanopterin dehydrogenase and F420‐dependent methylenetetrahydromethanopterin reductase) the energy metabolic $k_{\text{vivomax}}s$ did not appear much reduced from $k_{\mathrm{cat}}s$ and included also enzymes/subunits for which $k_{\mathrm{cat}}$ < $k_{\text{vivomax}}$ (Figure 3). Thus, energy metabolic $k_{\mathrm{cat}}s$ appeared underestimated, perhaps due to oxygen‐sensitivity of enzymes, and in vivo catalytic rates of energy metabolism show up as valuable descriptors of apparent enzymatic capacity of cells.

**FIGURE 4:**
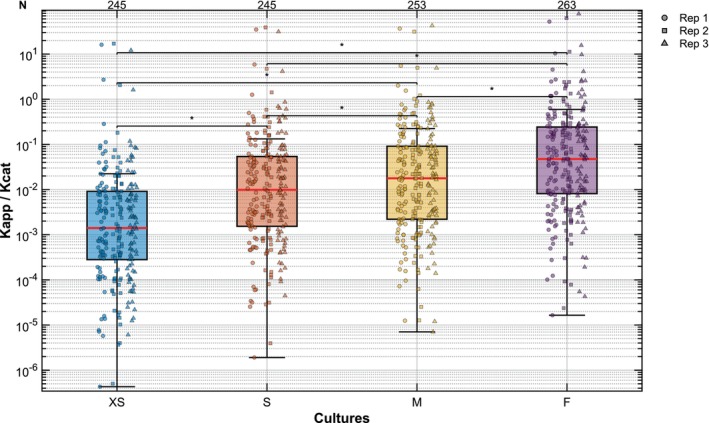
Impact of growth rate on the deviation of $k_{\mathrm{app}}$ from $k_{\mathrm{cat}}$. Boxplot of metabolic enzyme $k_{\mathrm{app}}/k_{\mathrm{cat}}$ (when $k_{\mathrm{app}}<k_{\mathrm{cat}}$) when *M. maripaludis* grew on formate at different dilution rates (XS = 0.003 h^−1^, *S* = 0.010 h^−1^, *M* = 0.028 h^−1^, *F* = 0.087 h^−1^) in chemostat cultures in three replicates (shapes: Circle, square and triangle distinguish replicates) in log‐scale with sample medians in red. Asterisk represents significantly different means (ANOVA and Tukey's test, *p value < 0.01 and *N* is the number of enzymes for which $k_{\mathrm{app}}/k_{\mathrm{cat}}$ could be determined for three cultures at each dilution rate).

Next, it was examined how the growth rate of *M. maripaludis* influenced in vivo catalytic rates of enzymes with respect to $k_{\mathrm{cat}}s.\;k_{\mathrm{app}}/k_{\mathrm{cat}}$ for enzymes of *M. maripaludis* growing in chemostats at four different dilution rates (i.e., XS = 0.003 h^−1^, S = 0.010 h^−1^, M = 0.028 h^−1^, F = 0.087 h^−1^) (Müller et al. 2021) revealed that as the dilution rate increased, $k_{\mathrm{app}}$ approached $k_{\mathrm{cat}}$ (Figure 4). $k_{\mathrm{app}}/k_{\mathrm{cat}}$ increased from XS to S (*p* value 1.65e‐12), from S to M (*p* value = 0.04906) and from M to F (*p* value 8.263e‐05), thus, by growth rate from the lowest growth rate to highest (ANOVA and Tukey's test, *n* = 245–263, *p* value < 0.05).

Enzymes operating closer to their maximal catalytic capacity under faster growth in chemostat was consistent with the growth rate being limited by the nutrient provision rate and not the enzymes' catalytic capacities. The faster nutrient provision likely increased enzyme saturation, thereby increasing η and elevating $k_{\mathrm{app}}$ toward $k_{\mathrm{cat}}$ while proteome allocation remained largely unchanged (Müller et al. 2021). Together with the $k_{\text{vivomax}}-k_{\mathrm{cat}}$ scatter analysis, this pattern supports strong modulation of the in vivo enzyme performance by metabolic state.

## 3 Discussion

In this study, in vivo catalytic rates of 99 metabolic enzymes of model methanogen *M. maripaludis* during growth on formate were determined and they were found to deviate from the corresponding $k_{\mathrm{cat}}$ more than previously reported for *E. coli* (Davidi et al. 2016) and *S. cerevisiae* (Chen and Nielsen 2021). This could be interpreted as *M. maripaludis* operating with lower enzyme saturation and/or closer to thermodynamic equilibrium than *E. coli* and *S. cerevisiae* , but the lower correlation could also reflect the growth in chemostats under growth‐limiting nutrient provision rate. Accordingly, as formate provision rate (and growth rate) increased (Müller et al. 2021) $k_{\mathrm{app}}$ of enzymes tended to move toward the generally higher $k_{\mathrm{cat}}$. Increase in $k_{\mathrm{app}}$ by growth rate requires growth rate dependent intracellular metabolite pools, previously reported in e.g., *E. coli* (Schaub and Reuss 2008).

For methanogenesis enzymes that operate with very small free‐energy changes (Thauer et al. 2008), the apparent in vivo catalytic rates may provide better estimates of catalytic capacity than $k_{\mathrm{cat}}$ values supported by in vitro assays. The enzymes are oxygen‐sensitive, e.g., MCR is irreversibly inactivated in the presence of oxygen as the reduced metal center and cofactors of MCR are easily oxidized (Costa and Whitman 2023), or use redox carriers whose balances are disturbed by oxygen. Consequently, predictive models trained on such data may systematically underestimate the true catalytic potential of these enzymes in vivo, allowing $k_{\text{vivomax}}$ to exceed predicted $k_{\mathrm{cat}}$ values.

Conclusions about the in vivo catalytic rates of *M. maripaludis* enzymes were enabled due to the wide scope of the underlying proteomic dataset (Müller et al. 2021). Quantitative proteomics detected and quantified 872 proteins, corresponding to ~51% of all the predicted proteins of *M. maripaludis* . The quantified proteins were strongly enriched with proteins associated with central metabolism, methanogenesis, ribosomal proteins and core biosynthetic functions. Thus, while low‐abundance regulatory or membrane‐associated proteins were underrepresented, which is typical for shotgun proteomics (Jiang et al. 2024), importantly for our determination of in vivo catalytic rates, the dataset captured the metabolic enzymes. In contrast, intracellular metabolite abundances of *M. maripaludis* have been reported to a narrower extent (Walker et al. 2012).

The in vivo catalytic rates of *M. maripaludis* enzymes can guide the efforts to engineer methanogens into hosts for industrial single carbon compound valorization to e.g., platform chemicals, fuels and material precursors. For instance, substantial overexpression of enzymes catalysing thermodynamically limited reactions may be required for increasing pathway flux (Noor et al. 2014). Accordingly, Thevasundaram et al. (2022) found the limitations in engineered polyhydroxybutyrate (PHB) production in *M. maripaludis* in redox cofactor availability and thermodynamic driving forces (Thevasundaram et al. 2022). Thus, such previous observations and our outcomes encourage focusing on methanogen host engineering to thermodynamic modelling guided optimal pathway designs.

## 4 Experimental Procedures

### 4.1 Data Sources

All simulations were performed using the mass‐ and charge‐balanced genome‐scale metabolic model of *M. maripaludis* , iMR539 (Richards et al. 2016).

Quantitative enzyme abundance data was obtained as relative proteome mass fractions (ppm) for *M. maripaludis* extra‐slow (XS), slow (S), medium (M) and fast (F) dilution rate chemostat cultures on formate in three biological replicates from (Müller et al. 2021).

Physiological data on formate uptake and methane rate was obtained from (Müller et al. 2021) for all dilution rates (XS, S, M and F) in three replicates. Turnover numbers ($k_{\mathrm{cat}},s^{-1}$) were retrieved from Metabolic Atlas (Lyu et al. 2025), in the current text we refer to these turnover numbers simply by $k_{\mathrm{cat}}$ or machine learning predicted $k_{\mathrm{cat}}$.

### 4.2 Protein Abundances

Relative protein mass fractions in parts per million (ppm) (Müller et al. 2021) were converted to abundances in cells in mmol gDW^−1^ using the molecular weight of protein and normalized by cell dry weight using experimentally determined protein content per gram cell dry weight in each culture (i.e., 58% XS1, 49% XS2, 51% XS3, 76% S1, 68% S2, 52% S3, 65% M1, 64% M2, 67% M3, 84% F1, 66% F2 and 70% F3) (Müller et al. 2021) using Equations (1) and (2).

(1) $$\mathrm{pm}\;\left(\mathrm{mg}\cdot \mathrm{gD}W^{-1}\right)=\frac{\mathrm{rmf}\left(\mathrm{ppm}\right)}{10^{6}}\cdot \mathrm{pc}\cdot 1000\;\left(\mathrm{mg}\right)$$

(2) $$\text{protein}\;\text{abundance in cells}\;(\text{mmol}\cdot gDW^{-1})=\frac{\text{pm(mg}\cdot gDW^{-1})}{MW(g\cdot mol^{-1})}$$

where pm is protein mass, rmf is relative protein mass fraction, pc is protein content per cell dry weight in culture and MW is molecular weight of protein.

Molecular weight of protein was determined by multiplying amino acid sequence length by average amino acid residue mass of 110 Da similarly to previous works (Milo 2013; Ata et al. 2026).

### 4.3 Growth Medium Definition for Simulations

GEM simulations were performed using a defined, formate‐based growth medium designed to mimic *M. maripaludis* cultivation without hydrogen provision. Formate served as the sole carbon source and electron donor. Formate uptake was enabled with a lower bound of −100 mmol·gDW^−1^·h^−1^. CO_2_ availability was maintained by allowing uptake with upper and lower bounds of 0 and −1000 mmol·gDW^−1^·h^−1^, respectively, and proton uptake was unrestricted. Nitrogen utilization was allowed exclusively as NH_3_, with a lower bound of −1000 mmol·gDW^−1^·h^−1^. Thus, N_2_ uptake and alanine uptake were disabled. Acetate uptake was also disabled.

### 4.4 Model Bounds and Kinetic Parameter Estimation

All simulations were performed using the *M. maripaludis* genome‐scale model iMR539 (Richards et al. 2016) with default bounds and biomass exchange flux as the objective function unless otherwise specified. For each of the four chemostat cultures (XS, S, M, F) in three biological replicates (Heirendt et al. 2019), formate uptake was constrained by setting the flux lower bound to the experimentally determined rate (Müller et al. 2021).

To estimate growth‐associated maintenance (GAM) coefficient, ATP hydrolysis term was temporarily removed from the biomass reaction by setting the stoichiometric coefficients of ATP, ADP and inorganic phosphate (Pi) to zero. For each chemostat culture replicate, the biomass exchange flux and formate uptake were fixed to experimental values (Table S1), the ATP hydrolysis reaction was maximized, and the associated ATP hydrolysis flux was recorded. Plotting ATP flux against the growth rate, GAM was determined as the slope of the fitted linear function. The GAM value was subsequently incorporated into the biomass reaction by adding the corresponding ATP hydrolysis requirement per unit of biomass produced.

Non‐growth‐associated maintenance (NGAM) was imposed through the ATP hydrolysis reaction (ATP → ADP + Pi). To identify the optimal NGAM value, a grid search over NGAM values ranging from 0 to 2.5 mmol·gDW^−1^ h^−1^ was conducted. For each candidate value, the ATP maintenance flux was fixed accordingly, and the model was simulated for each of the 12 cultures (Müller et al. 2021) by setting formate uptake to the experimentally determined rate. NGAM was determined as the value that minimized the total squared error between simulated and experimentally observed growth rates, while guaranteeing non‐zero growth rate predicted by model simulation for all experimental formate uptakes corresponding to non‐zero experimental growth rates. This condition was ensured through setting square error to infinite whenever the set NGAM value led to a model prediction of zero growth rate. This procedure yielded an NGAM, which was used in all subsequent analyses.

### 4.5 Model Modifications

Netflux directions of O‐succinylbenzoyl‐CoA 1,4‐dihydroxy‐2‐naphthoate lyase (rxn02898[c0]), isochorismate 2‐oxoglutarate cyclodienyltransferase (rxn04675[c0]), and hexaprenyl‐pyrophosphate synthase (rxn13477[c0]) were constrained to the direction of menaquinone synthesis. CODH (Carbon Monoxide Dehydrogenase) and ACS (Acetyl‐CoA Synthase) were flux‐coupled at a 1:1 ratio since CO is channelled in the enzyme complex from CODH to ACS (Biester et al. 2024). Netflux to the direction of formate oxidation was allowed for Formate dehydrogenase (Fdh) and heterodisulfide reductase (Hdr) (Halim et al. 2024). Mtd‐Hmd cycle was not allowed for H_2_ generation (Hendrickson and Leigh 2008).

### 4.6 Flux Variability Analysis of the Calibrated Model

Flux variability analysis (FVA) to assess the feasible ranges of fluxes.

FVA was conducted for each of the four cultures C (three biological replicates of each) in two steps:

1. Optimal growth: For each condition (defined by the medium composition and the specified formate uptake rate (Müller et al. 2021)), biomass exchange flux was maximized.
2. Near‐optimal flux ranges: Then, biomass exchange flux was constrained to the lower bound of 99% of the optimal value, and each flux was independently minimized and maximized to determine the feasible range.

For every biological replicate and flux range width $w=\left|v_{\max}-v_{\min}\right|$ was computed.

### 4.7 Quality Control of the Model: Agreement Between Simulations and Experimental Data

To assess the consistency of the calibrated model with experimental observations, condition‐specific, experimentally determined formate uptake rates, methane production rates and growth rates for *M. maripaludis* cultivated on formate (Müller et al. 2021) were complied. Using the previously determined GAM and the optimized NGAM value, the model was simulated at each chemostat condition (XS–F) and the model‐estimated methane exchange flux was recorded. Model's ability to reproduce experimental phenotypes was evaluated by setting, for each culture, formate uptake to the experimentally determined rate and maximizing biomass exchange flux.

### 4.8 Kinetic Capacity In Vivo, $k_{\mathrm{app}}$ and $k_{\text{vivomax}}$

$k_{\mathrm{app}}$ was determined for each enzyme in each culture C using Equation (3):

(3) $$\begin{array}{c} k_{{\mathrm{app}}_{c}}=\frac{v_{c}}{E_{c}}\; \end{array}$$

where $v_{c}$ is flux in mmol gDW^−1^ h^−1^ and $E_{c}$ protein abundance in mmol gDW^−1^. The maximum in vivo catalytic rate per enzyme, $k_{\text{vivomax}}$ was defined as the maximum $k_{\mathrm{app}}$ per enzyme over all the cultures C.

### 4.9 Correlation Analysis Between $k_{\text{vivomax}}$ and $k_{\mathrm{cat}}$

Machine learning predicted turnover numbers $k_{\mathrm{cat}}$ (Li et al. 2022) were obtained from Metabolic Atlas (Lyu et al. 2025). To assess the agreement between $k_{\mathrm{cat}}$ and in vivo catalytic rates $k_{\text{vivomax}}$, $k_{\text{vivomax}}$ and $k_{\mathrm{cat}}$ values were log‐transformed. Then, Pearson's coefficient (*R* ^2^) was calculated.

### 4.10 Treatment of Multimeric Enzymes Involved in Energy‐Metabolism

For multimeric enzymes involved in energy metabolism (i.e., methyl‐coenzyme M reductase (McrA/McrB/McrG), methyl‐H4MPT:HS‐coenzyme M methyltransferase (MtrE/MtrC/MtrH), formate dehydrogenase (FdhA/FdhB), heterodisulfide reductase (HdrA/HdrB2/HdrC2), formylmethanofuran dehydrogenase (Fwd/Fmd (A, C, D, F)) and F420‐non‐reducing [NiFe] hydrogenase (Vhu/Vhc (α, γ, δ))) $k_{\mathrm{app}}$ estimates were calculated for each subunit by using the abundance of a specific subunit and the model‐estimated flux of the corresponding reaction. Subunit stoichiometry of the complex or complex assembly was not accounted for.

### 4.11 Software

All processing, modelling, statistical testing and visualization were performed in MATLAB (R2019b). COBRA Toolbox v.3.0 (Heirendt et al. 2019) was used for metabolic model manipulations and setting up linear programming problems (i.e., model simulations). IBM ILOG CPLEX v.12.10 was used as a linear programming problem solver with feasTol = 1e−9.

## Author Contributions

**Enrique de Dios Mateos:** investigation, writing – review and editing. **Paula Jouhten:** conceptualization, funding acquisition, supervision, formal analysis, investigation, writing – original draft, writing – review and editing. **Silvan Scheller:** investigation, funding acquisition, writing – review and editing. **Hamza Faquir:** investigation, formal analysis, visualization, writing – original draft, writing – review and editing. **Celma Mekki:** formal analysis, visualization, writing – original draft, writing – review and editing, investigation.

## Funding

This work was supported by Jane ja Aatos Erkon Säätiö, 230053 and Business Finland, BFRK/2616/31/2023.

## Conflicts of Interest

The authors declare no conflicts of interest.

## Supporting information


**Figure S1:** Model calibration and validation.

**Table S1:** mbt270417‐sup‐0002‐TableS1.xlsx. *M. maripaludis* growth conditions and protein and enzyme data

## Data Availability

All data and code are available at https://version.aalto.fi/gitlab/microbial‐physiology‐public/kvivomethanococcusmaripaludis.
