# Supplementary figures and images for: In Vivo Enzyme Catalytic Rates in Formate‐Growing Methanococcus maripaludis
Source: Microb Biotechnol. 2026 Jul 24;19(7):e70417. doi: 10.1111/1751-7915.70417 (PMC13400843; doi:10.1111/1751-7915.70417)

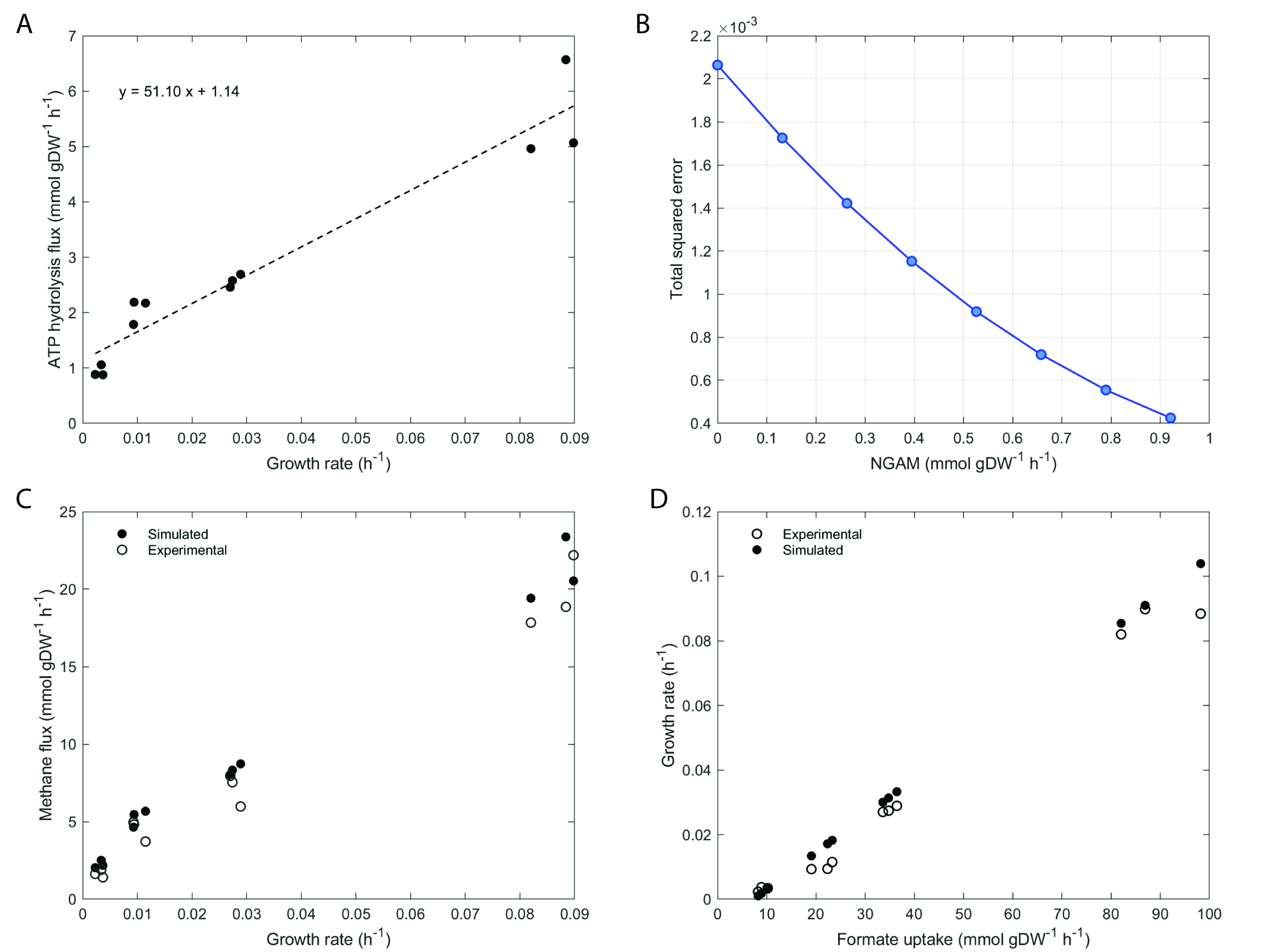

Supplement: Supplementary file 1 — Figure S1: Model calibration and validation. [file MBT2-19-e70417-s002.tif]
